# The Co-Existence of Obstructive Sleep Apnea and Bronchial Asthma: Revelation of a New Asthma Phenotype?

**DOI:** 10.3390/jcm8091476

**Published:** 2019-09-16

**Authors:** Angeliki Damianaki, Emmanouil Vagiakis, Ioanna Sigala, Athanasia Pataka, Nikoletta Rovina, Athina Vlachou, Vasiliki Krietsepi, Spyros Zakynthinos, Paraskevi Katsaounou

**Affiliations:** 1 Pulmonary Department “Saint George” General Hospital of Chania, 73300 Crete, Greece; angeldam@hotmail.com (A.D.); vikkikrietsepi@yahoo.gr (V.K.); 2 First ICU Clinic, National and Kapodistrian University of Athens, 10561 Athens, Greece; athvlachou@yahoo.com (A.V.); szakynthinos@yahoo.com (S.Z.); 3 Sleep Lab, First ICU Clinic Evangelismos Hospital, 10676 Athens, Greece; sleeplabathens@yahoo.gr (E.V.); giannasig@yahoo.com (I.S.); 4 Respiratory Failure Unit, G. Papanikolaou Hospital, Exohi, Aristotle University of Thessaloniki, 57010 Thessaloniki, Greece; patakath@yahoo.gr; 5 ICU, First Department of Pulmonary Medicine, Sotiria Hospital, 115 27 Athens, Greece; nikrovina@med.uoa.gr

**Keywords:** obstructive sleep apnea, bronchial asthma, alternative overlap syndrome

## Abstract

Bronchial asthma (BA) and obstructive sleep apnea (OSA) are common respiratory obstructive diseases that may coexist. It would be interesting to study the possible influence of that coexistence on both diseases. Until now, reviews focused mainly on epidemiology. The aim of this study was to review the literature in relation to epidemiology, pathophysiology, consequences, screening of patients, and treatment of the coexistence of OSA and BA. We pooled studies from the PubMed database from 1986 to 2019. OSA prevalence in asthmatics was found to be high, ranging from19% to 60% in non-severe BA, reaching up to 95% in severe asthma. Prevalence was correlated with the duration and severity of BA, and increased dosage of steroids taken orally or by inhalation. This high prevalence of the coexistence of OSA and BA diseases could not be a result of just chance. It seems that this coexistence is based on the pathophysiology of the diseases. In most studies, OSA seems to deteriorate asthma outcomes, and mainly exacerbates them. CPAP (continuous positive airway pressure) treatment is likely to improve symptoms, the control of the disease, and the quality of life in asthmatics with OSA. However, almost all studies are observational, involving a small number of patients with a short period of follow up. Although treatment guidelines cannot be released, we could recommend periodic screening of asthmatics for OSA for the optimal treatment of both the diseases.

## 1. Introduction

Bronchial asthma is a common inflammatory respiratory disease affecting up to 1–18% of the population in different countries. It is characterized by bronchial hyper-responsiveness (BHR) and presents with variable symptoms including wheezing, shortness of breath, chest tightness, and cough that vary according to disease severity [1]. The gold standard for the diagnosis is the improvement of forced expiratory volume in the first second (FEV_1_) >12% and 200 mL, 10–20 min after the inhalation of 100–200 mg of salbutamol [1].Almost 5–10% of asthmatics have severe, refractory asthma despite optimal therapy, and experience frequent exacerbations, hospital admissions, and healthcare utilization. They often use high doses of inhaled or oral steroids. A significant percentage of asthmatic patients, up to 70%, experience symptoms during sleep, and therefore have nocturnal asthma [2]. Both nocturnal symptoms in asthma and uncontrolled asthma have a profound effect on sleep quality [3]. The aim of asthma treatment is to achieve asthma control, which means having very few symptoms. Asthma control is assessed using validated questionnaires such as the asthma control questionnaire (ACQ) and the asthma control test (ACT) [1].

Obstructive sleep apnea (OSA) is the most frequent sleep-related breathing disorder. OSA with excessive daytime sleepiness ranged in frequency between 3% and 18% in men and 1–17% in women [4]. OSA is an under-diagnosed disorder [5] that is characterized by recurrent collapses of the upper airway during sleep, leading to a remarkable reduction or complete cessation of airflow despite ongoing breathing efforts. That airflow obstruction leads to repetitive hypoxia and fragmented sleep, and is therefore associated with hypertension, cardiovascular disease, diabetes, and stroke [6,7,8]. The gold standard diagnostic test is the overnight attended polysomnography (PSG), which is rather expensive and time-consuming. As a result, researchers often use validated questionnaires, such as the Berlin questionnaire (BQ), stop bang questionnaire (SBQ), and the sleep apnea scale of the sleep disorders questionnaire (SA–SDQ), to estimate the risk of having OSA [9,10,11]. The severity of OSA is determined using the apnea hypopnea index (AHI), which is defined as the number of apneas and hypopneas per hour of total sleep time (TST). Continuous positive airway pressure (CPAP) is the treatment of choice as it decreases long-term mortality [12].

OSA and BA share some common characteristics. First, they are both obstructive respiratory diseases, but with different mechanisms and anatomy of obstruction. A patient with BA and OSA has both upper and lower airway obstruction during sleep. Second, both diseases share common co–morbidities like obesity, allergic rhinitis, and gastro-esophageal reflux (GER). Patients with OSA and BA also have poor quality of sleep and may have increased morbidity and mortality. When they coexist, a bidirectional relationship may additionally affect each other. Since 2013, the coexistence of OSA and BA was defined as alternative overlap syndrome (AOS) to distinguish it from the overlap syndrome that is referred to as chronic obstructive pulmonary disease (COPD) and OSA [13]. While there are reviews in the literature, they mainly focused on the epidemiology. In order to determine the current state-of-the-art knowledge, we conducted a literature review, targeting epidemiology, pathophysiology, clinical consequences, screening, and treatment of AOS. We additionally aim to point out possible gaps for future research.

## 2. Methods

A review of the literature was performed pooling studies in English from the PUBMED database from 1986 to 2019. The following search terms were used: obstructive sleep apnea, bronchial asthma, and alternative overlap syndrome. We decided to focus on studies addressing the prevalence, diagnosis, pathophysiology, clinical outcomes, treatment, and screening of patients with obstructive sleep apnea and bronchial asthma (alternative overlap syndrome). During the search process, 673 articles emerged. Studies were excluded if not written in the English language or were carried out on a pediatric population (<18 years of age). Additionally, studies that were not relevant to our review were excluded. Finally, 92 articles were selected for this review.

## 3. Epidemiology

The vast majority of studies mainly referred to the prevalence of OSA in asthmatic populations. The prevalence was increased, ranging from 19% to 60% [14,15,16], and reaches up to 95% in severe BA in two studies [17,18], which suggests that the two diseases did not accidentally coexist. The great variability of this prevalence was the result of:

1. The diagnostic method used for OSA diagnosis. Early studies used questionnaires regarding OSA symptoms like habitual snoring, witnessed apneas, and daytime sleepiness [19,20,21,22]. Later, studies used validated questionnaires estimating the risk of having OSA [23,24,25]. Some used cardio-respiratory polygraphy [18,26], while a few used attended overnight polysomnography [14,27,28,29]. Other studies used a combination of the above-referred sleep studies [30,31,32].

2. The cut-offs used for the determination of OSA diagnosis. Some studies used AHI≥5 [14,33], others AHI≥15 [18,34], while others used the RDI (respiratory disturbances index includes apneas, hypopneas, and respiratory effort related arousals) [17].

3. The diagnostic method used for BA diagnosis. Few studies used spirometry or provocation tests [28,33,35]. The majority used questionnaires, where patients were asked to answer if they had asthma symptoms, receive asthma medications, or they have been informed about having asthma by their physicians [14,27,29,31].

4. The heterogeneity of the populations in relation to age, smoking status, and weight.

Early questionnaire-based, cross-sectional studies depicted a statistically significant higher prevalence of OSA symptoms, such as habitual snoring and witnessed apneas, in patients with BA as compared to the general population, independently of the body mass index (BMI), gender, age, or smoking status [19,20,21,22]. BA has been confirmed as an independent risk factor for habitual snoring, which is the mild end of the spectrum of sleep disordered breathing (SDB) [36].

In 2015, a large retrospective cohort study using data from the National Health Insurance of Taiwan, collected 38.840 newly diagnosed asthmatics between 2000 and 2010. Each patient matched to four people without asthma according to gender, age, and the date of the diagnosis. The occurrence of OSA was followed until the end of 2011. The overall incidence of OSA was 2.51-fold greater in the asthma cohort than in the comparison group (12.1 vs. 4.84 per person-years). Among asthmatics, the adjusted hazard ratio (HR) for OSA increased to 1.78 for asthma patients with one or less annual emergency room (ER) visits, and 23.8 for those who visited the ER more than once per year. Additionally, the adjusted HR in patients with inhaled steroids compared to those without receiving steroids was 1.33. The authors concluded that asthmatics had a greater risk for OSA, which was much greater when the asthma was more severe [33].

The same year, in another large study, this time a prospective population-based study, 547 selected subjects free of OSA at baseline (AHI < 5) on two consecutive overnight-attended PSG studies were followed every four years for OSA incidence. The BA diagnosis was based on questionnaires. Patients with asthma had a 39% increase in the risk of developing incident OSA as compared with controls, independently of the baseline covariates such as BMI, AHI, and BMI change over time. The risk was duration-dependent: for each 5-year increment in asthma duration, the risk for incident OSA increased by 7%. An asthma duration >10 years increased the risk by 65% [14].

In a recent meta–analysis of 26 studies (7675 patients), the prevalence/relative risk of OSA and the OSA risk (OSA diagnosis based on questionnaires) was 49.5%/2.64 and 27.5%/3.73, respectively, in asthmatics. Patients with BA and OSA had significantly greater BMI (average difference 2.15 kg/m^2^ *p* < 0.004), and sleepiness (average difference on the Epworth sleepiness scale, ESS = 3.98, *p* = 0.004) compared to asthmatics without OSA, while no difference was observed in FEV_1_ [16].

There is evidence in the literature that the prevalence of OSA in asthmatics is positively correlated with the severity of BA, ranging from 50 to 95% in severe asthma [17,18], and with the dosage of the received oral or inhalational steroids in a dose-dependent manner [17,37]. The possible association between the severity of BA and the severity of OSA remain controversial [18,28].

While the vast majority of studies address asthmatic populations, Alhabri et al. studied the relation between OSA and BA in the opposite direction. They found that patients with OSA diagnosed with PSG had a 35% increased prevalence of BA and these patients had a greater BMI, AHI, and the lowest oxygen saturation during sleep compared with OSA patients without BA. It seems like BA deteriorated OSA. Obesity (BMI > 35 kg/m^2^) was the only independent factor that could predict BA in OSA patients [27]. Increased prevalence of BA was also reported in OSA patients in some studies where the prevalence was not the primary endpoint. Kauppi et al., in a sample of 1586 patients with OSA, reported 13% having BA based on a questionnaire [31], while Wang et al. studying a sample of 466 patients in a sleep laboratory found that 16.5% had BA [35].

## 4. Pathophysiology

The exact mechanisms through which these diseases interact with each other are unknown, although much work has been done regarding this issue. The role of inflammation is a key contributor. Common co–morbidities like allergic rhinitis, obesity and GER also play an important role. Some of the proposed mechanisms are discussed below.

## 5. How BA Could Affect or Lead to OSA?

### 5.1. Mechanical Effects

Asthmatics with nocturnal asthma symptoms demonstrate hyperinflation during the day, meaning their functional residual capacity (FRC) is increased compared to healthy controls. However, the normal drop in FRC during sleep is exaggerated in asthmatics and specifically during Rapid Eye Movement (REM) sleep. This results in an increased airway resistance as an inverse relationship between lung volume and airway resistance is known to exist [38]. Moreover, Irvin et al. demonstrated that in asthmatics with nocturnal symptoms, there is an airway–parenchyma uncoupling, probably due to neural mechanisms, meaning that during the night, airway resistance is increased independently of lung volume [39]. Airway resistance could attenuate the normal tracheal tug inducing upper airway collapse [40].

### 5.2. Alterations in the Upper Airway Anatomy

The inflammatory infiltration of the upper airway in asthma [41], the increased fat deposition in the pharyngeal walls due to steroids use [17], or the presence of comorbidities such as obesity [42], lead to a diminished cross-sectional diameter of the upper airway. Moreover, allergic rhinitis, nasal polyps, and adenoids hypertrophy, which frequently accompany asthma, increase airflow resistance and create high negative pressure during inspiration that increase the risk of upper airway collapse [43,44].

Additionally, bronchial asthma possibly influences pharyngeal muscle function, either directly affecting neural sensor pathways because of inflammation or indirectly due to muscle weakness caused from the steroids that is the cornerstone of BA treatment [1]. Pharyngeal muscle myopathy increases collapsibility of the upper airway, increasing the risk of OSA. Yigla et al. reported a high prevalence of OSA (95%) among asthmatics receiving long-term chronic or frequent bursts of oral steroid therapy and indentified a positive dose–response relationship between steroids and OSA [17]. Other studies reported similar results [33,37]. The possible mechanisms through which asthma affects OSA are summarized from the authors in the figure below, although the impact of steroidsuse still remains controversial (Table 1).

## 6. How Could OSA Affect or Lead to BA?

The key factor seems to be the inflammation induced by OSA, both through the airway and systemic. The repeated episodes of a collapsed upper airway lead to hypoxia and eventually oxidative stress, which then cascades to systemic inflammation with increased serum levels of cytokines such as C-reactive protein, interleukin-6 (IL-6), and tumor necrosis factor (TNF-α). The distal airway inflammation seen in BA increases the probability of asthma attacks [45,46].

Additionally, in OSA, a neutrophil-predominant inflammation seems to begin from the nose, expands to the distal airways infiltrating the airway walls [47,48], and is related to the disease severity. Studies in experimental animal models have showed that the increased inspiration effort against the closed upper airway combined with intermittent hypoxia is associated with inflammation of the lungs [49,50].

Teodorescu et al. studied a large and objectively diagnosed sample of 255 asthmatic patients and estimated the risk of having OSA using the SA-SDQ questionnaire. A higher SA-SDQ score was associated with increased asthma symptoms, β_2_-agonist use, healthcare utilization, and worse asthma quality of life. They also conducted sputum induction and found higher percentages of neutrophils in asthmatics’ sputum with a high OSA risk compared to those without a high OSA risk (*p* = 0.001), whereas sputum eosinophil percentages were similar (*p* = 0.66). There was a significant association of SA-SDQ with sputum neutrophils. Namely, each increase in SA-SDQ by its standard deviation (6.85 units) was associated with a rise in sputum neutrophils of 7.78% (95% CI 2.33–13.22, *p* = 0.0006), independently of obesity and other confounding factors [24]. Authors concluded that OSA may be an important contributor to neutrophilic asthma.

The same results were also confirmed in 55 patients with severe asthma and OSA diagnosed using cardiorespiratory polygraphy [26]. Furthermore, by receiving bronchial biopsies with bronchoscopy, researchers found that the thickness of the bronchial basement membrane was negatively associated with the AHI [26]. Although more studies are needed, we may suggest that the coexistence of OSA in asthma patients may divert asthma inflammation to being neutrophilic, may contribute to airway remodeling, and eventually may result in a difficult-to-treat asthma.

Moreover, additional factors seen in OSA like GER, cardiac dysfunction, obesity, and increased levels of vascular endothelial growth factor (VEGF) and leptin have been proposed to increase BHR and deteriorate asthma outcomes [51,52,53,54].

**Figure 1:**
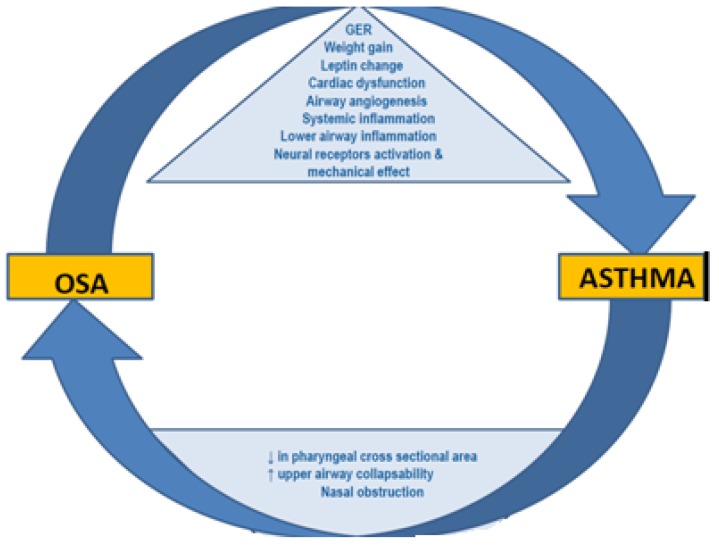
Pathophysiological mechanisms between Obstructive Sleep Apnea and Asthma. Adapted from Alkhalil et al. Sleep Medicine, 2009 [55].

The overall pathophysiologic mechanisms involved in the BA–OSA interaction are depicted in Figure 1.

## 7. Clinical Consequences of the Alternative Overlap Syndrome

There is strong clinical evidence to support the claim that OSA deteriorates BA outcomes.

Namely, the coexistence of OSA in asthmatics causes an increase in persistent nocturnal asthmatic symptoms compared with those without OSA [56,57,58]. Later, Teodorescu et al. investigated whether OSA was associated with daytime in addition to night-time asthma symptoms. Among 752 asthmatics, high OSA risk (using the SA-SDQ questionnaire) was associated with persistent daytime and night-time asthma symptoms (*p* < 0.0001 for each). A diagnosis of OSA was associated with both persistent daytime (*p* < 0.0001), in addition to night-time (*p* = 0.0008), asthma symptoms. The associations were retained in regression models where other known asthma aggravators, like obesity, were included. They concluded that unrecognized OSA may be the cause of persistent BA symptoms, both daytime and nocturnal [23].

OSA is the fifth independent risk factor (adjusted OR 3.4) among 13 clinical and environmental factors studied for recurrent exacerbations in difficult to treat asthma. The first four are psychological dysfunctioning, recurrent respiratory infections, GER, and severe chronic sinus disease [59]. Therefore, according to Global Initiative for Asthma (GINA) recommendations, chest physicians should investigate the possibility of underlying OSA in patients with difficult-to-treat asthma [1]. Moreover, in 2017, Wang et al. reported that the severity of OSA was independently correlated with the number of severe asthma exacerbations (OR = 1.32, *p* < 0.001) in a prospective cross–sectional cohort study [15].

Regarding asthma control, studies using ACT and ACQ questionnaires showed worse asthma control in asthmatics with OSA, although this was not statistically significant in all studies [25,60,61].

There is not much evidence addressing pulmonary function in asthmatic patients with OSA. The natural history of FEV_1_ decline in asthmatic patients has been reported to be 38–40.9 mL/year [62]. Known factors contributing to FEV_1_ decline in asthmatic patients are age, sex, smoking, acute exacerbations, obesity, and hypoxia [63,64,65]. In a recent retrospective study, using a sleep laboratory population with OSA patients, 77 asthmatic patients with OSA (diagnosed using PSG) were selected and followed for more than 5 years with spirometry. Asthmatic patients with OSA had substantially greater declines in FEV_1_per year compared to those without OSA in an AHI–dependent manner. Asthma patients with severe OSA (AHI > 30) had a decline in FEV_1_of 72.4 ± 61.7mL/year as compared to 41.9 ± 45.3 mL/year in those with mild to moderate OSA (5 < AHI ≤ 30) and 24.3 ± 27.5mL/year in those without OSA (AHI ≤ 5).The severity of OSA was the only independent factor for this decline after adjusting for other confounding factors like BMI, age, current smoking status, and number of emergency room visits/year. CPAP treatment significantly decreased the FEV_1_decline in the patients with severe OSA [35]. However, in a meta-analysis, in asthmatic patients with and without OSA, no significant difference was seen in FEV_1_ (mean difference = −2.28, *p* = 0.32) [16].

It is known that asthmatics have a low quality of sleep due to a variety of factors such as the increased frequency of nocturnal asthma, co-morbidities, bronchodilators, and corticosteroids [20,66]. As a result, they present excessive daytime sleepiness more frequently compared to controls [20]. For the first time in 2006 in a cross-sectional, clinic-based study investigating all these factors, Teodorescu et al. reported that sleepiness is common in asthmatics and may reflect occult OSA more often than the effects of asthma itself, other co-morbidities, or asthma medications [22]. Asthmatics with OSA have greater sleepiness than OSA-free asthmatics, scoring higher on the ESS [15,16].

The European Sleep Apnea Database (ESADA) cohort addressed the differences between asthmatics and non-asthmatics referred to sleep centers. An interesting finding was that asthmatic patients are under-referred for sleep studies, meaning that physicians are not aware or underestimate the coexistence of OSA and asthma in clinical practice. In that cohort, asthmatic women (with or without OSA) were more obese and reported more daytime sleepiness (according to ESS) than the non-asthmatic ones. This shows that obesity is the main factor for sleep referral in asthmatic women but may give implications about the existence of a specific phenotype as well [67].

There are very few reports investigating the objective parameters of sleep in subjects with AOS. In 2018, 384 adult women from the Sleep and Health Program in Sweden, an on-going community-based study, underwent overnight polysomnography. Women with both asthma and OSA had a longer sleep time in non-deep sleep stages N_1_and N_2_, and less time in the REM stage than the control group with no asthma or OSA. The group with BA and OSA had a lower mean oxygen saturation (93.4% vs. 94.7%, *p* = 0.004) than the group with OSA alone and spent more time with oxygen saturation below 90% than the patients with OSA only. The results remained after adjusting for age, BMI, and smoking status. BA was independently associated with lower oxygen saturation, while OSA was not. Authors concluded that coexisting OSA and BA is associated with poorer sleep quality and more profound nocturnal hypoxemia than either of the diseases alone [29]. Similar results concerning sleep architecture had already been reported in other studies [28,68].

While studies have highlighted OSA among asthma patients in outpatient settings, such data in the inpatient setting was sparse until 2015 when Becerra et al., using a 2009–2011 U.S. Nationwide Inpatient Sample, studied the impact of two common asthma co-morbidities—OSA and obesity—on the length and cost of hospitalization and on the need for invasive mechanical ventilation. They reported that OSA and obesity significantly increased the length of stay (OR = 1.07 in males and 1.14 in females, and 1.07 in males and 1.08 in females, respectively) and the total hospital charges in both genders (15% in males and 19% in females, and 8.6% in males and 9.6% in females, respectively). Furthermore, when they coexisted, this led to multiplied increases in the length of stay (OR = 1.19 in males and 1.24 in females) and hospital charges (24.9% in males and 28.5% in females).

OSA alone in asthmatics (OR = 2.56 in males and 3.22 in females) or in coexistence with obesity (OR = 2.85 in males and 3.60 in females) significantly increased the use of mechanical ventilation while obesity alone did not (OR = 0.97 in males and 1.01 in females) [69].

OSA and BA are associated with increased rates of systemic arterial hypertension [70,71], with inflammation being proposed as the basic pathophysiologic cause [72,73]. OSA in asthmatics lead to greater prevalence of arterial hypertension compared to asthmatics without OSA (adjusted OR = 2.20), as Ferguson et al. reported in a cross-sectional, questionnaire-based study. The authors concluded that the inflammation of the diseases act additionally [74].

The only study addressing the mortality in AOS is a retrospective study from South Korea. Kyu–Tae Han et al. used data from the National Health Insurance Service (NHIS) National Sample Cohort 2004–2013 in order to investigate the association between sleep disorders (International Classification of Diseases ICD–10: G.47) and mortality in patients with newly diagnosed BA (ICD–10: J.45) during outpatient care between 2004 and 2013. They excluded patients with sleep disorders diagnosed prior to the BA diagnosis. They studied asthmatic patients that were difficult to be controlled, had increased sleep disorders prevalence, and frequently used health services. The study sample consisted of 186.491 asthmatic patients newly diagnosed with asthma. A sleep disorders diagnosis that followed the asthma diagnosis and mortality was studied in these patients. A total of 5179 patients died during the study period (2.78%) because of lung cancer (10.5%), senility (6.1%), COPD (4.5%), myocardial infarction (4.4%), and stomach cancer (3.7%). Asthmatic patients with coexisting sleep disorders had an increased risk of death compared with asthmatics without sleep disorders after adjusting for confounding variables like age, sex, gender, income, and co-morbidities (hazard ratio [HR]: 1.451 (95% confidence interval [CI]: 1.253–1.681). The mean duration between the asthma diagnosis and death was shorter in asthmatics with sleep disorders (mean duration: 103.85 months), compared with asthmatics without sleep disorders (mean duration: 116.05 months, *p* < 0.0001). Authors concluded that the presence of sleep disorders in patients with asthma was associated with a high risk of mortality, as shown using Kaplan–Meier survival curves and a log rank test [75]. We summarize the findings concerning the impact of OSA on asthma in Table 2.

## 8. Treatment

Early CPAP application has shown a favorable effect on asthma parameters’ main symptoms. Most of these studies were retrospective, included a small number of subjects (9to16), having a short period of follow up (maximum 9 months), adherence to treatment was not based on objectives criteria, and there was a broad heterogeneity regarding the assessment of the results [56,57,58].

The first study investigating the impact of the long-term use of CPAP on asthma symptoms and on the control of the disease in a large number of patients was a questionnaire-based one. A survey questionnaire was distributed to all OSA patients using CPAP therapy in 2013. They received 1586 answers and 13% of them had BA. The mean duration of CPAP-usage was 5.7 years and their CPAP daily use was 6.3 h. Self-reported asthma severity (measured using a visual analogue scale) decreased significantly and the ACT score increased significantly from 15.35 to 19.8 (*p* < 0.001) without a significant change in the BMI [31].

The first prospective, multicenter study recently conducted in Spain examined asthma outcomes 6 months after CPAP use in 99 adult asthmatics with OSA. ACQ and the mini–asthma quality of life questionnaire (mini–AQLQ) was used for asthma control and quality of life, respectively, and objective measures of BA (spirometry) and OSA (PSG) as well. Both the control of the disease and the quality of life were significantly improved in general, but when the diseases were categorized according to their severity, only patients with moderate and severe BA and severe OSA, and patients who used CPAP >4 h/day, were significantly improved. There was a significant decrease in the percentage of asthma exacerbations seen in the six months after the use of CPAP (from 35.4% to7.2%, *p* = 0.015), a significant improvement in bronchial reversibility, symptoms of GER, rhinitis, and exhaled nitric oxide levels (eNO) (all *p* < 0.05). However, no significant changes were observed in the asthma medication used, drugs, body weight, and other asthma co-morbidities [32].

The favorable impact of CPAP on AOS has been attributed to the decrease of the local and systemic inflammation [76,77], and the improvement of both the heterogeneity of alveolar ventilation [78] and GER [79].

Τhere is one retrospective study that reported an improvement in FEV_1_ after CPAP treatment [35]. This finding was not confirmed in other studies [32,58,80], but the follow-up period was too short (maximum 6 months).

The impact of CPAP on BHR in patients with AOS is still a matter of controversy. Although most studies reported a decrease in BHR [81,82], this was not a constant finding [80]. However, more research is needed using a longer follow-up period in order to draw conclusions regarding the effect of CPAP treatment on pulmonary function.

According to current OSA guidelines, there is clear evidence in treating patients with moderate or severe OSA, whereas treatment should be restricted in mild OSA patients that are symptomatic or have serious co-morbidities, but still, asthma is not one of them. Considering asthmatic patients with mild OSA, there is no clear evidence regarding the best practice for treating these patients.

We therefore conclude that the optimal treatment practice for AOS is the best treatment of each disease separately and the recognition and treatment of the co-morbidities like obesity, GER. and more importantly allergic rhinitis, which is probably the basic reason of the high prevalence of OSA among the asthmatics. In a recent population-based study, a history of allergic rhinitis was associated with an increased risk of sleep-disordered breathing in the elderly and it was an independent risk factor of OSA in asthmatics (adjusted OR = 1.90, *p* = 0.046), while neck circumference was not (adjusted OR = 1.02, *p* = 0.654), although this is traditionally the most significant factor that predicts OSA [34].Patients who received intranasal corticosteroids had a significant improvement in OSA [83]. Another issue that needs investigation is the heterogeneity of the severity of both diseases in the same patient. Perhaps all the possible combinations may co-exist. Therefore, patients may have severe moderate or mild BA with severe, moderate, or mild OSA in any combination, as it happens with chronic bronchitis and emphysema in COPD. Consequently, treatment should be personalized.

We summarize the main studies addressing the effects of CPAP treatment for OSA on asthma outcomes in Table 3.

The role of alternative treatment in OSA like uvulopalatopharyngoplasty, oral appliances, upper-airway stimulation, or surgical interventions on AOS has not been investigated yet. Bariatric surgery is known to improve asthma control, pulmonary function, BHR, and quality of life. Bariatric surgery also improves OSA parameters [84,85]. The effect of bariatric surgery on AOS has not been studied yet.

The effect of biological asthma treatments and leukotriene inhibitors have not been studied regarding AOS.

## 9. Screening

From the above-mentioned data it is obvious that BA is a risk factor for OSA occurrence and development, and OSA might aggravate BA reciprocally. OSA worsens asthma outcomes [25,59,69]. On the other hand, there is strong evidence that long-term use of CPAP in patients with BA and OSA improves symptoms, asthma control, and quality of life [31,32]. That is why the need for screening asthmatics for OSA is imperative. Apart from the overnight-attended PSG, which is an expensive and time-consuming method, and therefore, it is inconvenient for screening a large number of subjects, there is a gap in the literature regarding the use of OSA questionnaires in asthmatic populations. For the first time, Huan et al. compared the SBQ with BQ in 123 objectively diagnosed asthmatics undergoing overnight-attended PSG. Compared with BQ, SBQ had a higher diagnostic sensitivity (84.4% vs. 60%), lower specificity (79.5% vs. 91%), lower positive predictive value (70.4% vs. 79.4%), and higher negative predictive value (90% vs. 80%) to detect moderate-to-severe OSA at an AHI cut-off of 15/h. They concluded that SBQ is the preferable sleep questionnaire over BQ for detecting the risk of having moderate-to-severe OSA in asthmatics [86].

## 10. Summary

OSA has been found to have increased prevalence, ranging from 19 to 60%, in asthmatic patients, and even reaching 95% in severe asthma cases. The diagnosis of OSA has been made either using a questionnaire or an overnight-attended PSG. Home sleep apnea testing (HSAT) is likely to be much more accurate than questionnaires and better for identifying moderate to severe OSA, but it is not recommended in patients with severe cardiorespiratory disease and there are no special recommendations for asthmatic patients [87,88].Given that patients with AOS usually suffer from severe asthma, and therefore studies using HSAT would not have included them, we did not focus on HSAT in the current review. However, we included studies that have evaluated asthmatic patients using HSAT and PSG, as seen in Table 1 (Sundbom et al.) and Table 2 (Kauppi et al., Serrano Pariente et al.). It is difficult to accurately estimate the prevalence of AOS based on the fact that the prevalence of OSA depends on the definition used and the population studied, and ranges between 2% and 10% in women and between 4% and 31% in men [89,90,91]. More specifically, while the Wisconsin study found a prevalence of sleep-disordered breathing (SDB) defined as an AHI≥5/hr to be 9% in women and 24% in men [89], in a more recent systematic review, the overall prevalence for AHI ≥5 events/h was found to range from 9% to 38% and was higher in males and in some elderly groups, and it increased reaching 90% in men and 78% in women. For AHI≥15 events/h, the prevalence ranged from 6% to 17% in the general population, reaching 49% in the advanced ages. OSA prevalence increases with obesity and age [92].

The prevalence of OSA in asthmatics is positively correlated with the severity and duration of asthma and the dosage of oral or inhaled steroids. It seems that asthma is a risk factor for the OSA occurrence, but in order for a causal relationship to confirmed, large prospective studies are needed with objective pulmonary function and sleep measurements.

As the impact of OSA on asthma causes a neutrophilic inflammation, contributes to airway remodeling, increases nocturnal and diurnal persistent symptoms, worsens sleep quality and nocturnal hypoxemia increasing sleepiness the next day, deteriorates asthma outcomes, is an independent risk factor for asthma exacerbations, and increases the days of hospitalization and health resource utilization, OSA should not be seen as a simple co-morbidity. AOS is a new asthma phenotype that should be uncovered. Additionally, although it is clear that the co-existence of asthma and OSA increases asthma exacerbations, the association of the number of exacerbations with sleep parameters like AHI, ODI (oxygen desaturation index), and AI (arousal index) has not yet been defined.

Finally, since we lack data from double-blind studies, there are currently no available treatment guidelines for the combination of OSA and asthma, and the treatment of asthma and OSA is limited to each specific disease’s guidelines. In Table 4, we outline the major take away points, as well as areas where further research is needed.

GINA guidelines urge physicians to look for OSA in asthmatics [1]. However, there are limitations in diagnosing OSA in a primary care setting, which may lead to the under-diagnosis of OSA. This problem is worse when asthma coexists because of overlapping nocturnal symptoms. Although SBQ has not been validated in large asthmatic populations, there is an urgent need to use it periodically forasthmatics, especially in those with refractory asthma, obesity, allergic rhinitis, and GER. These patients will need to be tested for OSA at the sleep lab. Early diagnosis of OSA in asthmatic patients will lead to the appropriate treatment with CPAP and will stop the vicious circle of OSA worsening asthma control and escalating asthma pharmaceutical treatment.

We conclude that there is an urgent need for the early diagnosis of OSA in asthmatic patients in order to effectively treat both diseases and lessen the economic burden.

## Figures and Tables

**Table 1:** The effect of asthma on Obstructive Sleep Apnea.

Attenuation of the Normal Tracheal Tug/Alteration in the Upper Airway Anatomy	
Diminished cross-sectional diameter of the upper airway due to Asthma inflammation	Nasal obstruction due to Nasal polyps
Increased fat deposition in the pharyngeal walls due to: Sleep deprivation Depression Exercise limitation Obesity Steroids use	Adenoids hypertrophy
Upper airway muscle dysfunction Steroid use Disruption of sensory neural pathways due to inflammation	Allergic rhinitis

**Table 2:** Impact of Obstructive Sleep Apnea (OSA) on asthma.

Study	Study Design	Population	Asthma Diagnosis	OSA Diagnosis	Results
Teodorerscu, M et al. J Asthma 2012 [23]	Cross-sectional	*N* = 828 subjects with BA Allergy and pulmonary clinics	ATS guidelines	SA-SDQ and review of medical notes	High OSA risk associated with persistent daytime (OR = 1.96, 95% CI = 1.31–2.94) and night-time (OR = 1.97, 95% CI = 1.32–1.94) asthma symptoms.
Wang et al. Sleep Med 2016 [15]	Prospective cross-sectional cohort study	*N* = 146 asthmatics *N* = 157 controls Asthma follow-up in outpatient clinics	Physician diagnosis	PSG	Annual number of severe asthma exacerbations was significantly higher in the OSA group compared to the no-OSA group (*p* < 0.001). AHI significantly correlated with the number of exacerbations (*p* < 0.001).
Tay, T.R Respirology 2016 [60]	Cross-sectional	*N* = 90 asthmatics	Specialist physician diagnosis (76 had variable airflow obstruction)	Clinical symptoms and BQ or previous positive PSG	OSA or high OSA risk in 35/90 (38.9%). Univariate analysis showed asthmatics with OSA to have worse ACT (*p* = 0.034) and worse AQLQ (*p* = 0.029), but not in multivariate analysis.
Kim et al. Ann Allergy Asthma Immunol 2013 [25]	Cross-sectional	*N* = 217 asthmatics Controls = 0 Randomly recruited from tertiary care clinic	1. Airway reversibility with FEV_1_ > 12% and 200 mL post SABA or positive metacholine provocation test 2. Persistent symptoms 3. Physician diagnosis of asthma (need all three)	BQ	A total of 89/217 (41%) were high risk for OSA. The high OSA risk group had a lower ACT score than the low OSA risk group but it was not statistically significant: 20.9 ± 3.6 vs. 21.5 ± 3.3 (*p* = 0.091).
Teodorescu et al. Chest 2010 [61]	Cross-sectional	*N* = 472 asthmatics from tertiary care clinic visits	Asthma or allergy specialist using ATS guidelines and ACQ for BA control	SA-SDQ	A total of 109/472 (23%) were high risk for OSA. High OSA risk associated with 2.87-fold higher odds for having poorly controlled asthma (*p* = 0.0009, 95% CI = 1.54–5.32).
Wang et al. BMC Pulm Med 2017 [35]	Retrospective	*N* = 77 asthmatics Sleep lab of a tertiary hospital	ATS criteria. Airway reversibility with FEV_1_ > 12% and 200 mL post SABA or average daily diurnal peak flow variability was more than 10%. Regular follow up with pulmonary function tests at least every six months for more than 5 years.	PSG	The decline in FEV_1_ among asthmatics with severe OSA (AHI > 30/h) was 72.4 ± 61.7 mL/year (*N* = 34), as compared to 41.9 ± 45.3 mL/year (*N* = 33, *p* = 0.020) in those with mild to moderate OSA (5 < AHI ≤ 30) and 24.3 ± 27.5 mL/year (*N* = 10, *p* = 0.016) in those without OSA (AHI ≤ 5).
Teodorescu et al. Sleep Med 2006 [22]	Cross-sectional	*N* = 115 asthmatics Routine asthma follow-up visits	Physician diagnosis	SA-SDQ	ESS associated with SA-SDQ (*p* < 0.0001) and asthma severity step (*p* = 0.04), but was not associated with asthma severity step in multiple regression analysis.
Sundbom et al. J Clin Sleep Med 2018 [29]	Cross-sectional	Women pooled from the Sleep and Health program in Sweden. *N* = 36 patients with BA *N* = 15 patientswith BA + OSA *N* = 109 patients with OSA	Positive answers to either of the following questions: 1. Have you an attack of asthma in the last 12 months? 2. Are you currently taking any medicine, including inhalers, aerosols, or tablets for asthma?	Full-night home PSG	Women with BA+OSA had a longer sleeping time in N1 and N2 sleep stages than the control group with no BA or OSA. They had also lower mean oxygen saturation (93.4% vs. 94.7%, *p* = 0.04) than the women with OSA alone. The results were consistent after multivariate analysis. BA was independently associated with lower oxygen saturation while OSA was not.
Becerra et al. Respiratory Medicine 2016 [69]	Retrospective	2009–2011 U.S Nationwide Inpatient Sample International Classification of Diseases, 9th Revision, Clinical Modification (ICD–9–CM) 493.x to identify primary hospitalizations for asthma. *N* = 179.789 primary BA hospitalizations	Secondary diagnosis code for BA hospitalizations with comorbid conditions of obesity (ICD–9–CM 278.0x) and OSA (ICD–9–CM 327.23)	Secondary diagnose code for OSA (ICD–9–CM 327.23) objectively based OSA diagnosis	Increased hospital length of stay was associated with the presence of obesity (OR for males = 1.07, OR for females = 1.08), OSA (OR for males = 1.07, OR for females = 1.14), and both obesity and OSA (OR for males = 1.19, OR for females = 1.24). Increased total hospital charges was related to obesity (8.64% for males and 9.61% for females), OSA (15.39% for males and 19.13% for females), and both co-morbidities (24.94% for males and 28.50% for females). Presence of OSA alone increased the odds of needing mechanical ventilation for males (OR = 2.56) and females (OR = 3.22), as did presence of both co-morbidities (OR for males = 2.85, OR for females = 3.60).
Ferguson et al. Lung 2014 [74]	Cross-sectional, questionnaire-based	*N* = 812 asthmatics at routine follow-up at allergy and pulmonary clinics	ATS criteria, managed by an academic specialist	SA-SDQ	Hypertension was diagnosed in 191 asthmatics (24%), OSA in 65 (8%), and OSA or high OSA risk (combined OSA variable) in 239 (29%). With adjustment for covariates, associations with hypertension remained significant for some FEV_1_% categories (70–79% odds ratio = 1.60 [95% CI: 0.90–2.87]; 60–69% OR = 2.73 [95% CI = 1.28–5.79]; < 60% OR = 0.96 [95% CI = 0.43–2.14]), and for OSA (OR = 2.20 [95% CI = 1.16–4.19]).
Han et al. BMC Pulmonary Medicine 2016 [75]	Retrospective	National Health Insurance Service (NHIS) National Sample Cohort 2004–2013 in South Korea. A total of 186.491 patients who were newly diagnosed with BA during the study period at outpatient care were followed for OSA development and mortality.	ICD–10: J.45	ICD–10:G.47 only when it followed a BA diagnosis	A total of 5179 (2.78%) patients died during the study period. Sleep disorders in patients previously diagnosed with asthma were associated with a higher risk of mortality (hazard ratio (HR): 1.451 (95% CI = 1.253–1.681). The mean duration between BA diagnosis and death was shorter in asthmatics with sleep disorders (mean duration = 103.85 months) compared to asthmatics without sleep disorders (mean duration = 116.05 months, *p* < 0.0001)

BA = Bronchial Asthma, OSA = Obstructive Sleep Apnea, ATS = American Thoracic Society, SA-SDQ = Sleep Apnea of Sleep Disorders Questionnaire, PSG = Polysomnography, AHI = Apnea Hypopnea Index, OR = Odds Ratio, CI = Confidence Interval, HR = Hazard Ratio, BQ = Berlin Questionnaire, ACT = Asthma Control Test, AQLQ = Asthma Quality of Life Questionnaire, SABA = Short Acting B Agonist, ACQ = Asthma Control Questionnaire, FEV_1_ = Forced Expiratory Volume in the first second, ESS = Epworth Sleepiness Scale.

**Table 3:** Effects of C–PAP treatment for OSA on asthma outcomes.

Study Design	SAMPLE SIZE	Main Characteristics	C–Pap Treatment/Adherence	Method for OSA Diagnosis	Changes With C–Pap
Chan et al., single-center, control-CPAP, off-cCPAP Am Rev Resp Dis 1988 [56]	9 subjects	Severe asthma, AHI 21.1/h	2 weeks, no objective adherence	PSG	Improved symptoms, reduced bronchodilator use, improved AM and PM pre-/post-bronchodilator PEFR that paralleled the treatment period and returned to pretreatment levels during the CPAP-off period
Guilleminault et al., single-center Eur Resp J 1988 [57]	10 subjects	BA with moderate to severe obstruction (FEV_1_ 54% predicted), RDI = 51/h	6–9 months, no objective adherence	PSG	Improved symptoms
Ciftsi et al., single center Resp Med 2005 [58]	16 subjects	Nocturnal asthma, AHI 44/h	2 months, no objective adherence	PSG	Improved symptoms, no response in FEV_1_
Lafond et al., single center Prospective Eur Resp J 2007 [80]	20 subjects	Stable asthma of various control levels, AHI = 48/h	6 weeks, objective adherence. CPAP use 6.7 h per night (subjects excluded if CPAP use < 4h per night).	PSG	Improved mini-AQLQ scores. No improvement in BHR (metacholine challenge) No changes in FEV_1_
Kauppi et al., single center retrospective cross-sectional questionnaire study Sleep Breath 2006 [31]	152 subjects using CPAP in 2013	Self-reported physician-diagnosed BA, REI (respiratory event index) > 41.2/h	5.7 years, daily use 6.3 h, objective adherence	Home polygraphy	Decreased self-reported BA severity, improved ACT scores, reduced percentage of patients using rescue medication daily, no changes in BMI
Wang et al., single center, retrospective BMC Pulm Med 2017 [35]	Subset of 13 subjects	BA based on symptoms and spirometry Severe OSA AHI>30/h	2 years, objective adherence, CPAP use 6.4 h/night	PSG	Reduced annual decline in FEV_1_ A trend toward reduced ER visits (*p* = 0.058)
Serrano Pariente et al., multicenter (15 centers), prospective Allergy 2017 [32]	99 subjects	BA: Intermittent: 11%, mild persistent:17%, moderate persistent:48%, severe persistent: 24%, RDI = 46.3/h	6 months, objective adherence recorded and non–compliant subjects were not excluded	PSG: 30% of patients or cardiorespiratory polygraphy: 70%	Improved symptoms; improved ACQ and mini-AQLQ scores in asthmatics with severe OSA (AHI > 30), as well in asthmatics with moderate to severe BA; improved ACQ and mini AQLQ scores in subjects who used CPAP > 4 h/night; reduced proportion of patients without a well-controlled asthma score; reduced bronchodilator use; reduced exacerbations; reduction in proportion of patients with positive bronchodilator response; improved symptoms of GER and rhinitis; improved exhaled nitric oxide values; no changes in FEV_1_

OSA = Obstructive Sleep Apnea, C-pap = Continuous positive airway pressure, AHI = Apnea Hypopnea Index, h = hour, PSG = Polysomnography, AM = Morning, PM = Afternoon, PEFR = Peak Expiratory Flow Rate, BA = Bronchial Asthma, FEV_1_ = Forced Expiratory Volume in the first second, RDI = Respiratory Disturbances Index, AQLQ = Asthma Quality of Life Questionnaire, BHR = Bronchial Hyper-responsiveness, REI = Respiratory Events Index, ACT = Asthma Control Test, BMI = Body Mass Index, ER = Emergency Room, ACQ = Asthma Control Questionnaire, GER = Gastro-Esophageal Reflux.

**Table 4:** Coexistence of Obstructive Sleep Apnea in asthmatics patients.

Section	Conclusions	Future Research Needed
Epidemiology	OSA is quite common among asthmatics, so a new asthma phenotype seems to emerge.	
Diagnosis	Clinicians should suspect OSA in asthmatic patients with the above characteristics: obesity sleepiness allergic rhinitis GER severe uncontrolled asthma lasting over a decade requiring high doses of steroids	
Pathophysiology	OSA and asthma interact in terms of pathophysiology AOS inflammation is neutrophilic AOS is a difficult-to-treat asthma phenotype There is a possible causal relationship between asthma and OSA	The exact pathophysiologic mechanisms remain unknown More studies are needed to confirm neutrophilic inflammation in asthmatic patients with OSA Large prospective studies with objective measures of pulmonary function and sleep are needed for a causal relationship to be confirmed
Clinical consequences	Increased night-time and daytime symptoms Increased asthma exacerbation (related to AHI) Worse asthma control Deterioration of sleep quality (increased sleep time of non-deep sleep stages (N1, N2), reduced sleep time in deeper stages (REM), excessive sleepiness the next day); it is associated with profound hypoxemia during sleep Increased annual FEV_1_ decline (related to OSA–severity) Increased arterial hypertension Increased hospitalization days and costs, especially when associated with obesity Increased need of mechanical ventilation Increased mortality (possible)	The association between the number of asthma exacerbations and objective sleep parameters must be investigated More studies are needed regarding sleep architecture and objective parameters like ODI, AI, and heart rate Large prospective long-term studies are needed to show how pulmonary function is affected in asthmatics with OSA More research is needed in order to study mortality, hypertension, hospital admissions, and hospitalization requirements
Treatment	C-PAP treatment seems to have a favorable effect on asthma outcomes, where it: Improves daytime and night–time symptoms Reduces asthma exacerbations Improves asthma control Improves quality of life Reduces BHR (still controversial) Lessens annual FEV_1_ decline Improves GER and rhinitis symptoms Reduces eNO levels	Large prospective randomized controlled trials are needed to confirm the beneficial effects of C-PAP treatment on AOS The effectiveness of alternative OSA treatments and asthma medications on AOS have to be explored There is a gap in literature concerning the need of C-PAP treatment in asthmatics with mild OSA
Screening of patients with AOS	The need for screening asthmatics presenting with the described phenotype is imperative SBQ is recommended If SBQ is indicative for OSA, patients must be referred to a sleep lab as well if the risk of having OSA remain high and SBQ is not indicative	More studies are needed in large asthmatic populations to confirm the diagnostic value of SBQ and investigate if other questionnaires have the potential to accurately estimate the risk of having OSA

OSA = Obstructive Sleep Apnea, GER = Gastro Esophageal Reflux, AOS = Alternative Overlap Syndrome, AHI = Apnea Hypopnea Index, REM = Rapid Eye Movement, FEV_1_ = Forced Expiratory Volume in the first second, ODI = Oxygen Desaturation Index, AI = Arousal Index, C-PAP = Continuous positive airway pressure, BHR = Bronchial Hyper-responsiveness, eNO = exhaled Nitric Oxide, SBQ = Stop Bang Questionnaire.

## Abbreviations

ΑΗΙ	Apnea Hypopnea Index
ACQ	Asthma Control Questionnaire
ACT	Asthma Control Test
AI	Arousal Index
AM	Morning
AOS	Alternative Overlap Syndrome
AQLQ	Asthma Quality of Life Questionnaire
BMI	Body Mass Index
BA	Bronchial Asthma
BHR	Bronchial Hyper-responsiveness
BQ	Berlin Questionnaire
CI	Confidence Interval
COPD	Chronic Obstructive Pulmonary Disease
CPAP	Continuous Positive Airway Pressure
CRP	C-Reactive Protein
e-NO	exhaled- Nitric Oxide
ER	Emergency Room
ESS	Epworth Sleepiness Scale
FEV1	Forced Expiratory Volume in One Second
FRC	Functional Residual Capacity
GER	Gastro-Esophageal Reflux
GINA	Global Initiative for Asthma
HSAT	Home Sleep Apnea Testing
HR	Hazard Ratio
ICD–10	International Classification of Diseases
IL–6	Interleukin–6
Mini–AQLQ	Mini Asthma Quality of Life Questionnaire
OSA	Obstructive Sleep Apnea
ODI	Oxygen Desaturation Index
OR	Odds Ratio
PEFR	Peak Expiratory Flow Rate
PM	Afternoon
PSG	Polysomnography
REI	Respiratory Events Index
REM	Rapid Eye Movement
RDI	Respiratory Disturbances Index
SABA	Short Acting B Agonist
SBQ	Stop Bang Questionnaire
SA-SDQ	Sleep Apnea of Sleep Disorders Questionnaire
SDB	Sleep Disordered Breathing
TNF	Tumor Necrosis Factor
TST	Total Sleep Time
VEGF	Vascular Endothelial Growth Factor
